# 

**Published:** 1887-04

**Authors:** 


CONTENTS.

Vol. 34 No. 4.

PAGE.

The Occult Forces ............... 73
Injurious and Adulterated Beverages ............... 77
The Prayer Cure Practically Applied ............... 80
Henry Ward Beecher ............... 81
Quinine ............... 82
Food Adulterations ............... 83
Hygiene, Its Agency in Therapeutics ............... 84
Drugs and Medicines ............... 86
Surgery Extraordinary ............... 87
Dental Surgery ............... 88
A Strange Story from Chicago ..... 89
Evils of Water Drinking ............... 90
Rheumatism ............... 91
Book Notices ............... 92
Household ............... 93
Miscellaneous ............... 95

PAGE.

INDEX TO ADVERTISEMENTS OF HALF A PAGE AND OVER.

Winchester's Hypophosphate .. I
Lactated Food ............... II
Celerina ............... III
Lactopeptine ............... IV
Castoria ............... IV
Horsford's Acid Phosphate ... V
Empire Syringe Co. ............... VI
Bovinine ............... VII
Ten Valuable Books Free ...... VIII
Crystalline Phosphate .......... VIII
Hollow Suppositories, 2d page cover
Dr. Carter Moffats' Ammoniaphone, ............... 3d page cover
Goodyear Rubber Co. 3d page cover

